# Acceptance and Commitment Therapy for people living with motor neuron disease: an uncontrolled feasibility study

**DOI:** 10.1186/s40814-023-01354-7

**Published:** 2023-07-07

**Authors:** Rebecca L. Gould, Charlotte Rawlinson, Ben Thompson, Kirsty Weeks, Rebecca Gossage-Worrall, Hannah Cantrill, Marc A. Serfaty, Christopher D. Graham, Lance M. McCracken, David White, Robert J. Howard, Matt Bursnall, Mike Bradburn, Ammar Al-Chalabi, Richard Orrell, Suresh K. Chhetri, Rupert Noad, Aleksandar Radunovic, Tim Williams, Carolyn A. Young, David Dick, Vanessa Lawrence, Laura H. Goldstein, Tracey Young, John Ealing, Hamish McLeod, Nicola Williams, Helen Weatherly, Richard Cave, Theresa Chiwera, Francesco Pagnini, Cindy Cooper, Pamela J. Shaw, Christopher J. McDermott, and the COMMEND Collaboration Group, Annmarie Burns, Caroline Dancyger, Annily Dee, Susie Henley, Mark Howell, Naoko Kishita, Selina Makin, Emily Mayberry, Mark Oliver, Alexandra Richards, Emma Robinson, Liz Tallentire

**Affiliations:** 1 Division of Psychiatry, University College London, Wing B, 6th Floor Maple House, 149 Tottenham Court Rd, London, W1T 7NF UK; 2 Clinical Trials Research Unit, School of Health and Related Research, University of Sheffield, Sheffield, UK; 3 Priory Hospital North London, London, UK; 4 Strathclyde Psychology, Department of Psychological Sciences & Health, University of Strathclyde, Glasgow, UK; 5 Department of Psychology, Uppsala University, Uppsala, Sweden; 6 Maurice Wohl Clinical Neuroscience Institute, King’s College London, London, UK; 7 Department of Clinical and Movement Neurosciences, Institute of Neurology, University College London, London, UK; 8 Lancashire Teaching Hospitals NHS Foundation Trust, Lancashire, UK; 9 Department of Neuropsychology, Derriford Hospital, Plymouth, UK; 10 Barts Health NHS Trust, London, UK; 11 The Newcastle Upon Tyne Hospitals NHS Foundation Trust, Newcastle Upon Tyne, UK; 12 The Walton Centre NHS Foundation Trust, Liverpool, UK; 13 Norfolk and Norwich University Hospital, Norwich, UK; 14 Health Services & Population Research Department, Institute of Psychiatry, Psychology & Neuroscience, King’s College London, London, UK; 15 Department of Psychology, Institute of Psychiatry, Psychology & Neuroscience, King’s College London, London, UK; 16 School of Health and Related Research, University of Sheffield, Sheffield, UK; 17 Manchester Centre for Clinical Neurosciences, Salford Royal NHS Foundation Trust, Salford, UK; 18 Mental Health and Wellbeing, University of Glasgow, Glasgow, UK; 19 Primary Care Clinical Trials Unit, Oxford University, Oxford, UK; 20 Centre for Health Economics, University of York, York, UK; 21 Language and Cognition, University College London, London, UK; 22 Department of Psychology, Università Cattolica del Sacro Cuore, Milan, Italy; 23 Sheffield Institute for Translational Neuroscience, University of Sheffield, Sheffield, UK

**Keywords:** Motor neuron disease, Acceptance and Commitment Therapy, Feasibility, Acceptability, Psychological health

## Abstract

**Background:**

Motor neuron disease (MND) is a fatal, progressive neurodegenerative disease that causes progressive weakening and wasting of limb, bulbar, thoracic and abdominal muscles. Clear evidence-based guidance on how psychological distress should be managed in people living with MND (plwMND) is lacking. Acceptance and Commitment Therapy (ACT) is a form of psychological therapy that may be particularly suitable for this population. However, to the authors' knowledge, no study to date has evaluated ACT for plwMND. Consequently, the primary aim of this uncontrolled feasibility study was to examine the feasibility and acceptability of ACT for improving the psychological health of plwMND.

**Methods:**

PlwMND aged ≥ 18 years were recruited from 10 UK MND Care Centres/Clinics. Participants received up to 8 one-to-one ACT sessions, developed specifically for plwMND, plus usual care. Co-primary feasibility and acceptability outcomes were uptake (≥ 80% of the target sample [*N* = 28] recruited) and initial engagement with the intervention (≥ 70% completing ≥ 2 sessions). Secondary outcomes included measures of quality of life, anxiety, depression, disease-related functioning, health status and psychological flexibility in plwMND and quality of life and burden in caregivers. Outcomes were assessed at baseline and 6 months.

**Results:**

Both a priori indicators of success were met: 29 plwMND (104%) were recruited and 76% (22/29) attended ≥ 2 sessions. Attrition at 6-months was higher than anticipated (8/29, 28%), but only two dropouts were due to lack of acceptability of the intervention. Acceptability was further supported by good satisfaction with therapy and session attendance. Data were possibly suggestive of small improvements in anxiety and psychological quality of life from baseline to 6 months in plwMND, despite a small but expected deterioration in disease-related functioning and health status.

**Conclusions:**

There was good evidence of acceptability and feasibility. Limitations included the lack of a control group and small sample size, which complicate interpretation of findings. A fully powered RCT to evaluate the clinical and cost-effectiveness of ACT for plwMND is underway.

**Trial registration:**

The study was pre-registered with the ISRCTN Registry (ISRCTN12655391).

**Supplementary Information:**

The online version contains supplementary material available at 10.1186/s40814-023-01354-7.

## Key messages regarding feasibility

- To the authors' knowledge, No study to date has evaluated the feasibility and acceptability of Acceptance and Commitment Therapy (ACT) for people living with motor neuron disease (plwMND)
- A priori indicators of success and outcomes related to feasibility and acceptability indicated that: i) it is possible to recruit plwMND to a study of ACT for improving psychological health; and ii) ACT appears to be acceptable to this population, as demonstrated by initial engagement, session attendance and satisfaction with the intervention.
- The feasibility findings highlight that a fully powered randomised controlled trial (RCT) of ACT for improving psychological health in plwMND is justified. They further suggest that the main issues to consider in an RCT include minimising drop out, examining maintenance of effects at follow-up, and exploring ways in which results can be generalised to a broader population.

## Background

Motor neuron disease (MND) is a fatal, progressive neurodegenerative disease that predominantly affects motor neurons in the motor cortex and spinal cord, causing progressive weakening and wasting of limb, bulbar, thoracic and abdominal muscles. There is no cure for MND, and median survival is approximately 2–3 years following symptom onset, with only 4–10% surviving more than 10 years [1–3]. Furthermore, riluzole, the sole disease-modifying drug licensed in the UK, prolongs median survival for just 2–3 months at 1 year [4].

As a consequence of the nature and impact of MND symptoms and the poor prognosis, people living with MND (plwMND) and their families are faced with numerous psychological challenges, in addition to physical, social and financial difficulties. These include uncertainty due to variability in the disease course, cumulative losses in multiple domains that require continual psychological adjustment, and feelings of isolation due to a lack of awareness of MND [5, 6]. Given these challenges, it is not surprising that some plwMND experience psychological distress during the disease course. Prevalence rates of up to 44% for depression and 30% for anxiety have been reported [7–9], with rates varying depending on assessment measures used, and higher in those with bulbar onset MND [10, 11]. Psychological distress in plwMND is associated with a range of negative outcomes, including shorter survival times, poorer quality of life and increased risks of suicide and mortality [12–16]. However, clear evidence-based guidance on how psychological distress should be managed in this population is lacking.

Current recommendations for managing psychological distress in plwMND are limited due to a lack of evidence to support such recommendations [17, 18]. Previous systematic reviews of psychological interventions to reduce psychological distress and improve psychological wellbeing in plwMND have highlighted limited research of varying quality [19, 20]. For example, a randomised controlled trial (RCT) of meditation training compared to usual care in 100 plwMND reported promising results with respect to quality of life, depression and anxiety, but was limited by high attrition rates (57% and 71% at 6- and 12-month follow-up, respectively) [21]. Other studies were limited by small sample sizes, lack of a control group and/or lack of follow-up assessment. Consequently, previous reviews have concluded that there is insufficient evidence to recommend specific psychological therapies for plwMND and that more research is urgently needed.

The evolution of behavioural and cognitive therapies thus far is considered to have occurred in three waves [22]: the ‘first wave’ of therapies (such as behavioural therapy) focus on direct behavioural change. The ‘second wave’ of therapies (such as traditional or conventional cognitive behavioural therapy) focus on directly changing the form or frequency of one’s internal experiences (e.g. thoughts, emotions, physical sensations, etc.). In contrast, the ‘third wave’ of therapies (such as Acceptance and Commitment Therapy and mindfulness-based interventions) focus on changing how one relates to these internal experiences, rather than attempting to control them.

Acceptance and Commitment Therapy (ACT) may be particularly suitable for people with life-limiting illnesses and disabling long-term conditions such as MND, muscle disorders, brain injury and chronic pain [5, 23–25]. ACT is an acceptance-based behaviour therapy [26] that has a strong evidence base in chronic pain, while the evidence base in other physical and mental health conditions is growing [27]. For example, there is preliminary evidence that ACT may be beneficial for improving psychological wellbeing in other neurodegenerative conditions, including multiple sclerosis and Parkinson’s disease [28, 29].

ACT uses acceptance, mindfulness, motivational and behaviour change techniques to reduce unhelpful attempts to control, change or eliminate internal experiences (such as negative thoughts, emotions and physical sensations) and increase engagement in life-enriching activities. These techniques include helping people to be more: i) open to and accepting of their internal experiences rather than engaging in ineffective or futile struggles with them; ii) aware of their experiences and focused on the here-and-now rather than ruminating about the past or worrying about the future; and iii) committed to doing things guided by what really matters to them rather than by experiences they want to avoid.

To the authors' knowledge, no study to date has evaluated ACT in plwMND. Consequently, the primary aim of this uncontrolled study was to examine the feasibility and acceptability of ACT for improving the psychological health of plwMND. A secondary aim was to obtain preliminary estimates of 'signals of efficacy' of ACT for improving psychological health in plwMND.

## Materials and methods

All reporting is in accordance with Consolidated Standards of Reporting Trials (CONSORT) [28] and Template for Intervention Description and Replication (TIDieR) [29] guidelines. CONSORT and TIDieR checklists are provided in Additional Files 1and 2 and additional methodological information is presented in Additional File 3. Ethical approval was granted by the London-Dulwich Research Ethics Committee (18/LO/0227).

### Design

This was a pre-registered, uncontrolled, feasibility study (ISRCTN Registry ISRCTN12655391).

### Participants

PlwMND and their caregivers were recruited from 10 UK MND Care Centres/Clinics. Eligible plwMND were aged ≥ 18 years with a diagnosis of definite, laboratory-supported probable or probable familial or sporadic Amyotrophic Lateral Sclerosis (ALS, which is diagnostically synonymous with MND [30]) using the World Federation of Neurology’s El Escorial criteria [31]. Eligible caregivers were aged ≥ 18 years and were the primary caregiver of the person living with MND.

Exclusion criteria for plwMND included:

1. Need for gastrostomy feeding or non-invasive ventilation i.e. those in stages 4A or 4B of the King’s College London clinical staging system [32], as these are markers of significantly reduced life expectancy and more advanced disease stage (and hence an indicator that participants might not survive the duration of the study);
2. Diagnosis of dementia using standard diagnostic guidelines [33, 34];
3. Currently receiving ongoing formal psychological therapy delivered by a formally trained psychologist or psychotherapist or unwilling to refrain from engaging in such formal psychological therapy during the receipt of ACT;
4. Insufficient understanding of English to enable engagement in ACT and completion of screening measures and patient-reported outcome measures;
5. Lacking capacity to provide fully informed written consent, verbal consent (for those who cannot provide written consent), or consent via the use of a communication aid;
6. Need for treatment for severe psychiatric disorder such as schizophrenia or bipolar disorder, or those expressing suicidal ideation with active plans/suicidal behaviours and intent;
7. Other medical factors that could compromise full study participation such as intellectual disabilities or severe sensory deficits.

### Procedure

Potential participants were identified and approached about the study through local clinicians, clinical and research databases, and community advertisements. Participants who provided informed consent (either written, verbally or via a communication aid) and met eligibility criteria were invited to participate. Participation in the study for plwMND involved engagement in therapy sessions and completion of outcome measures. Participation in the study for caregivers involved an invitation to attend up to three key therapy sessions (as outlined in the next section), with the consent of the person living with MND, and completion of outcome measures. All plwMND and study therapists were also invited to participate in semi-structured qualitative interviews to explore feedback in relation to delivery and receipt of the intervention. Qualitative findings will be reported elsewhere.

### Intervention

We previously made a series of recommendations as to how to adapt psychological interventions for the specific psychological, physical, communication and cognitive needs of plwMND [5]. These recommendations were based on a systematic examination of individuals' priorities and concerns [5], and a manualised ACT intervention focused on the person living with MND was developed based on these findings. The intervention comprised up to eight one-to-one sessions of ACT, supported by online audio recordings, with each session up to one hour in duration. Sessions were delivered in person within the outpatient clinic or participant's home or via video call, depending on participant preference and therapist availability. The first six sessions were weekly, and subsequent sessions were fortnightly and then monthly to facilitate sessions ending. All participants living with MND received usual multidisciplinary care in addition to ACT.

Although the intervention was focused on the person living with MND, caregivers were invited to attend the assessment session and two sessions focused on committed action, with the consent of the person living with MND. The purpose of this in the assessment session was to ensure that those involved in the care of the person living with MND were on board with the aims of ACT (‘living better’ rather than ‘feeling better’). The purpose of this in the committed action sessions was to ensure that goals involving assistance from the caregiver were set collaboratively between the person living with MND and the caregiver. If requested by the person living with MND, the caregiver was able to attend all therapy sessions as an observer rather than active participant in therapy.

All sessions, except the first and last ones, followed the same structure. Sessions commenced with a short mindfulness exercise designed to increase awareness of the present moment. This was followed by brief ratings of how much the participant had been trying to change or get rid of difficult thoughts, feelings and sensations, how much they had been worrying about the future or dwelling on the past, and how much they had been living a life guided by what was important and really mattered to them (i.e. their values and goals). A brief assessment of suicidal ideation, including any plans, intent and protective factors, if necessary, was conducted next. Following this, there was a recap of the concepts and issues discussed in the previous session, as well as a discussion of the participant's experience of completing the home practice. The remainder of the session was spent broadly addressing a key ACT process, together with associated skills, metaphors, experiential exercises and home practice tasks, as outlined in Table 1. However, therapists were encouraged to bring other ACT processes into each session too (e.g. by asking process-specific questions), where appropriate, so that they could respond flexibly to what was being discussed in the session (so called "dancing around the hexaflex"). See Additional File 4 for information about the core psychologically inflexible processes and their psychologically flexible counterparts, as well as examples relevant to plwMND. The pace of the sessions could be modified by the therapist, depending on the participant's needs and abilities, as therapists had a choice about which and how many metaphors and experiential exercises could be delivered in each session. The session ended with a summary of what had been discussed in the session, as well as a discussion of that week’s home practice.

**Table 1:** An outline of the ACT sessions, together with associated metaphors, experiential exercises and home practice

Session	Main focus of the session (with metaphors and/or experiential exercises)	Home practice
1	*Aim:* Assessment of current issues, goals of therapy and introduction to ACT *Exercises:* Introducing ACT *Online supplemental material:* Introducing ACT audio file	1) Notice the things that are important and matter to you and the thoughts, feelings and sensations that get in the way of this
2-7^a^	*ACT process:* Values *Aim:* Clarify what is important and matters to you (i.e. what you want to be doing and how you want to be doing that) *Exercises:* Centering exercise; Lifetime achievement award, Values List or Values Questions; Life compass *Online supplemental material:* Small steps exercise audio file	1) Notice the thoughts, feelings and sensations that get in the way of the things that are important and matter to you, and what you do when they show up 2) Take the smallest step that would move you towards one of your values
	*ACT process:* Acceptance *Aim:* Explore workability of emotional control strategies and introduce an alternative to emotional control *Exercises:* Centering exercise; Passengers on the bus; Accepting all of you or Physicalising exercise *Online supplemental material:* Willingness exercise audio file	1) Notice the thoughts, feelings and sensations that you would have to be willing to have in order to move towards the things that are important and matter to you 2) Take the smallest step that would move you towards one of your values
	*ACT process:* Defusion and contact with the present moment *Aim:* Practice skills for unhooking from thoughts, feelings and sensations in order to move towards the things that are important and matter to you *Exercises:* Centering exercise; "I notice I'm having…”, Singing the thought or saying it in a silly voice, Writing the thought in different colours/different styles/reverse order, “Milk, milk, milk” or Imagine a thought on a computer screen; Notice 5 things or Tracking your thoughts in time *Online supplemental material:* Leaves on a stream audio file	1) Practice unhooking yourself or stepping back from your thoughts, feelings and sensations in order to move towards the things that are important and that matter to you 2) Take the smallest step that would move you towards one of your values
	*ACT process:* Self-as-context *Aim:* Practice skills for noticing the distinction between you and your thoughts, feelings and sensations in order to move towards the things that are important and matter to you *Exercises:* Centering exercise; Labels exercise, Very brief self-as-observer and/or House/furniture metaphor *Online supplemental material:* Connecting with the noticing you audio file	1) Practice looking at your thoughts, feelings and sensations, including the stories that you tell about yourself, in a different way, from a different viewpoint in order to move towards the things that are important and matter to you 2) Take the smallest step that would move you towards one of your values
	*ACT process:* Committed action *Aim:* Explore external barriers and ways of overcoming them using selection, optimisation and compensation principles *Exercises:* Centering exercise; Part 1 of willingness and action plan incorporating selection, optimisation and compensation principles *Online supplemental material:* Your kind friend audio file	1) “Find another route around external barriers” in order that you can continue moving towards the things that are important and matter to you 2) Take the smallest step that would move you towards one of your values
	*ACT process:* Committed action *Aim:* Set goals and actions in service of values *Exercises:* Centering exercise; Part 2 of willingness and action plan incorporating selection, optimisation and compensation principles *Online supplemental material:* Problem solving for external problems	1) Set and publicly commit to completing your goals and steps in service of your values 2) Take the smallest step that would move you towards one of your values
8	*Aim:* Review skills and concepts discussed and the metaphors and/or exercises used to illustrate them; review gains made in the sessions; and explore ways of more effectively handling thoughts, feelings and sensations in the future *Exercises:* Centering exercise *Online supplemental material:* Hexaflexercise audio file	N/A

^a^The order of sessions 2–7 was chosen by the therapist, depending on the participant's needs and according to the individualised ACT case conceptualisation developed for each participant

Therapists were qualified clinical psychologists, counselling psychologists or Cognitive Behavioural Therapists, with a minimum of one year's experience in delivering psychotherapy interventions. Therapists attended a 4-day experientially based ACT training workshop, which was developed and delivered by members of the research team with experience of ACT. This included training on: MND symptoms, prognosis and treatment; working with augmentative and alternative communication devices; psychological issues in MND; ACT core processes; ACT assessment and case conceptualisation; and adapting ACT for plwMND. ACT competency was established through an ACT Knowledge Questionnaire [35] and a clinical vignette-based quiz, developed as part of the training package. Weekly telephone group supervision was provided throughout the study delivery period by two clinical psychologists and a psychiatrist, with a minimum of five years’ experience of ACT. Therapists were invited to attend on at least a fortnightly basis.

### Usual care

All participants received usual multidisciplinary care in addition to ACT comprising standard care as outlined in NICE Clinical Guideline NG42 for MND [17]. This included medication for managing MND and MND-related symptoms, treatments for MND-related symptoms (e.g. physiotherapy, non-invasive ventilation and gastrostomy), equipment and adaptations to aid activities of daily living, communication and mobility, and access to other services (including clinical psychology and neuropsychology, counselling, social care, respiratory ventilation, palliative care gastroenterology, orthotics, mobility/assistive technology/communication equipment services and community neurological care teams).

### Treatment fidelity

All therapy sessions were recorded using encrypted digital voice recorders and uploaded to a secure network server. Ten percent of sessions were randomly selected (stratified by phase of study recruitment and intervention and therapist) and assessed for treatment fidelity by two independent ACT therapists using the ACT Treatment Integrity Coding Manual (ACT-TICM) [36]. This comprises 14 items, rated on a scale from 1 (not at all) to 5 (extensively), which assess ACT components, anti-ACT components (i.e., such as encouraging attempts to control, change, avoid or eliminate uncomfortable thoughts and feelings), general assessment, overall adherence to the manual and overall therapist competence. Independent raters also provided feedback in relation to what therapists did well and what they could have done differently with respect to ACT. Assessment of treatment fidelity using the ACT-TICM occurred regularly throughout the study so that therapists could receive feedback on their intervention delivery.

### Data collection

A range of socio-demographic and clinical data were collected at screening and baseline (0 months), including the Edinburgh Cognitive Behavioural ALS Screen (ECAS) [37] and the Motor Neuron Disease Behavioural Instrument (MiND-B) [38]. Outcome measures were completed at baseline and 6 months via face-to-face interview, telephone or post. This time period was chosen in order to account for variability in disease prognoses.

### Outcomes

The co-primary outcomes and a priori indicators of success were uptake (≥ 80% of the target sample [*N* = 28] recruited over the recruitment period) and initial engagement with the intervention (≥ 70% completing at least 2 sessions), which were pre-agreed with the Funder based on their commissioning brief [39]. Secondary outcomes included additional measures of acceptability and feasibility: satisfaction with therapy at 6 months using the Satisfaction with Therapy and Therapist Scale-Revised (STTS-R) [40]; failure to recruit and attrition due to lack of acceptability of the intervention; referral rate; and failure to recruit and attrition for reasons other than lack of acceptability of the intervention.

Secondary patient-reported outcome measures at baseline and 6 months were the McGill Quality of Life Questionnaire-Revised (MQOL-R) [41], Hospital Anxiety and Depression Scale modified for plwMND (mHADS) [42, 43], Acceptance and Action Questionnaire-II (AAQ-II) [44], EQ-5D-5L (including the Visual Analogue Scale [VAS]) [45], ALS Functional Rating Scale-Revised (ALS FRS-R) [46], Client Service Receipt Inventory (CSRI) [47] modified for plwMND, and non-physical adverse events and physical self-harm. Caregiver-reported outcome measures at baseline and 6 months were the EQ-5D-5L (plus VAS) and Zarit Burden Interview (ZBI) [48]. See Additional File 3 for further details.

### Data analyses

Categorical measures were summarised using frequencies and percentages, while continuous measures were summarised using means and standard deviations (SDs) or medians and interquartile ranges (IQRs) for very skewed distributions. No formal data analysis was conducted, as recommended in pilot and feasibility studies [49, 50]. However, change scores across time were calculated for individuals who had observations at both baseline and 6-months, and then averaged across individuals. Cohen's *d* effect sizes (with accompanying confidence intervals) were also calculated by dividing the mean change score by the SD of the change scores, as previously recommended for paired data [51]. Finally, Reliable Change Index (RCI) scores [52] were calculated in order to examine whether any changes in outcome measures across time were reliable (i.e. greater in magnitude than could be explained by measurement error or artefacts of repeated measurement), based on published estimates of internal consistency [41, 44, 53–57].

### Sample size

A sample size of 28 plwMND from 10 recruitment sites, assuming 20% attrition at 6 months [58], allowed engagement with the intervention to be estimated to within a standard error of 10%. This sample size was consistent with sample sizes of 24–35 participants conventionally recommended for pilot and feasibility studies [59–61].

## Results

### Study flow

**Fig. 1:**
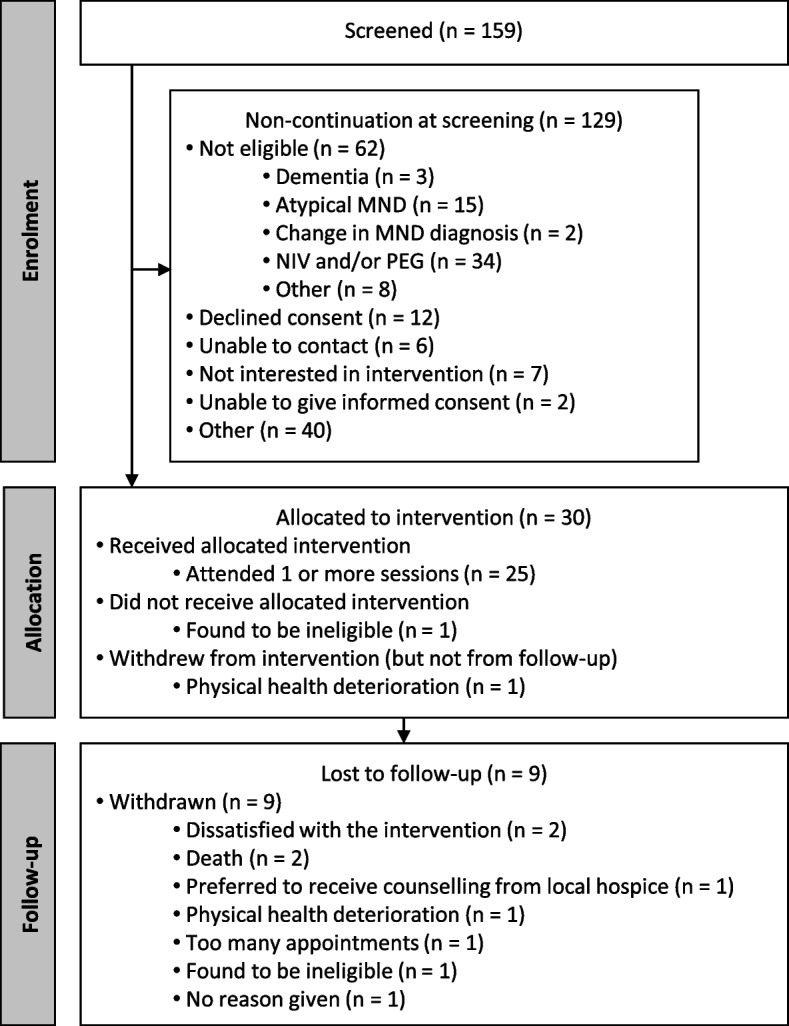
Summary of recruitment and follow-up of participants in the study

As shown in Fig. 1, 159 potential participants were referred to the study in July-November 2018, and 6-month follow-ups were conducted in January-May 2019. Thirty plwMND consented to participate in the study, with one participant later being found to be ineligible. Eight participants were lost to follow-up (not including the participant who was found to be ineligible), with four dropping out before receiving any therapy sessions ( three due to physical health or death and one due to preferring counselling). Eighteen plwMND had a caregiver who consented to participate in the study (the rest either did not have a caregiver or did not have a caregiver who consented to participate).

### Baseline characteristics

Baseline demographic and clinical characteristics are described in Tables 2 and 3. Of note, only a small proportion of participants reported a comorbid diagnosis of depression (5/29, 17%) or suicidal ideation (5/29, 17%), while none reported a comorbid diagnosis of anxiety. However, a third of participants (34%, 10/29) reported being prescribed psychotropic medication at baseline, eight of which were for mood-related reasons (though only three of these reported a diagnosis of depression).

**Table 2:** Demographic characteristics of plwMND and caregivers

	**plwMND (*N* = 29)**		**Caregivers (*N* = 18)**	
**Variable**	**N (missing N, %)**	**Mean (SD) or N (%)**	**N (missing N, %)**	**Mean (SD) or N (%)**
Mean age (years)	29 (0, 0%)	58.4 (13.8), range 31–75	15 (3, 17%)	58.6 (14.9), range 29–77
Sex	29 (0, 0%)		15 (3, 17%)	
Female		14 (48%)		8 (53%)
Male		15 (52%)		7 (47%)
Marital status	29 (0, 0%)		16 (2, 11%)	
Co-habiting		2 (7%)		1 (6%)
Divorced		3 (10%)		1 (6%)
Married		20 (69%)		13 (81%)
Other		1 (3%)		0 (0%)
Single		2 (7%)		1 (6%)
Widowed		1 (3%)		0 (0%)
Ethnicity	29 (0, 0%)		15 (3, 17%)	
Asian/Asian British		0 (0%)		0 (0%)
Black/Black British		1 (3%)		0 (0%)
Mixed		0 (0%)		1 (7%)
White/White British		28 (97%)		14 (93%)
Other		0 (0%)		0 (0%)
Mean years of education	28 (1, 3%)	14.3 (3.8), range 9–21	14 (4, 22%)	13.9 (3.7), range 9–18
Employment status	29 (0, 0%)		15 (3, 17%)	
Paid work		8 (28%)		6 (40%)
Voluntary work		1 (3%)		1 (7%)
Retired		12 (41%)		5 (33%)
Not working		8 (28%)		2 (13%)
Other		0 (0%)		1 (7%)

*SD* Standard deviation. One participant who was recruited but later found to be ineligible is not included here

**Table 3:** Clinical characteristics of plwMND

Variable	N (missing N, %)	Mean (SD), median (IQR) or N (%)
Probable or definite MND	26 (3, 10%)	
Amyotrophic Lateral Sclerosis		19 (73%)
Progressive Muscular Atrophy		1 (4%)
Progressive Bulbar Palsy		1 (4%)
No MND variant specified		5 (19%)
Median months since diagnosis	27 (2, 7%)	9.0 (25.0), range 0.7–107
Median months since symptom onset	26 (3, 10%)	27.5 (38.4), range 3–166
No. prescribed riluzole	28 (1, 3%)	19 (68%)
ECAS^a^	29 (0, 0%)	
Mean total score (possible range 0–136)		111.9 (12.9), range 82–129
Mean ALS-specific total score (possible range 0–100)		83.4 (11.4), range 55–97
MiND-B mean total score (possible range 9–36)^b^	23 (6, 21%)	33.1 (3.8), range 24–36
No. with a self-reported comorbid physical health diagnosis	29 (0, 0%)	
Yes		18 (62%)
No		11 (38%)
No. with a self-reported comorbid mental health diagnosis	29 (0, 0%)	
Depression		5 (17%)
Anxiety		0 (0%)
Suicidal ideation	29 (0, 0%)	
Yes		5 (17%)
No		24 (83%)
No. prescribed psychotropic medication	29 (0, 0%)	10 (34%)^c^
Amitriptyline		2 (10%)
Citalopram		5 (25%)
Escitalopram		1 (5%)
Fluoxetine		1 (5%)
Sertraline		1 (5%)

One participant who was recruited but later found to be ineligible is not included here

*ALS* Amyotrophic lateral sclerosis, *ECAS* Edinburgh Cognitive Behavioural ALS Screen, *IQR* Interquartile range, *MiND-B* MND Behavioural Instrument, *SD* Standard deviation

^a^Higher scores indicate fewer cognitive symptoms

^b^Higher scores indicate fewer behavioural symptoms

^c^No participant was prescribed more than one psychotropic medication

### Session delivery

The mean number of sessions attended was 5.5 (SD 3.4; median 8.0, IQR 6.5), with 59% (17/29) attending all 8 sessions. The median waiting time for therapy was 3.3 weeks (IQR 2.6).

### Primary outcomes

Both of the a priori targets for uptake and initial engagement with the intervention were met: 104% (29/28) of the target sample were recruited and 76% (22/29) completed at least 2 sessions.

### Secondary outcomes

#### Acceptability

Mean scores on the STTS-R at 6 months were high (see Table 4): 79% (15/19) and 100% (19/19) of participants rated therapy and therapists as "satisfactory" (i.e. scoring ≥ 21/30), respectively. The majority of participants (79%, 15/19) rated therapy as making things somewhat or a lot better, with none rating therapy as making things somewhat or a lot worse. Few potential participants were screened and not recruited due to not being interested in ACT (7/159, 4%), and few recruited participants were lost to follow-up due to dissatisfaction with it (2/29, 7%).

**Table 4:** Mean scores, mean change scores and effect sizes at baseline and 6 months

	**Baseline**		**6 months**		**Change score (baseline-6 months)**			
**Outcome measure**	**N**	**Mean (SD)**	**N**	**Mean (SD)**	**N**	**Mean (SD)**	**ES**	**95% CI**
*plwMND (N* = *29)*								
Quality of life: MQOL-R								
Global (possible range 0–10)	29	6.8 (2.2)	21	7.0 (1.7)	21	0.24 (1.79)	0.13	-0.30 to 0.56
Physical (possible range 0–10)	29	5.8 (2.2)	21	5.5 (1.8)	21	0.67 (1.83)	0.36	-0.08 to 0.80
Psychological (possible range 0–10)	29	7.0 (2.6)	21	7.6 (2.1)	21	-0.12 (1.87)	-0.06	-0.49 to 0.37
Existential (possible range 0–10)	29	6.7 (2.3)	21	7.1 (1.8)	21	-0.12 (1.50)	-0.08	-0.51 to 0.35
Social (possible range 0–10)	29	8.2 (1.9)	21	8.6 (1.5)	21	-0.30 (1.50)	-0.20	-0.63 to 0.23
Total score (possible range 0–10)	29	6.9 (1.9)	21	7.2 (1.5)	21	0.03 (1.36)	0.02	-0.41 to 0.45
Mood: mHADS^a^								
Depression (possible range 0–18)	26	3.4 (3.2)	21	3.0 (2.6)	18	0.06 (2.44)	0.02	-0.44 to 0.48
Anxiety (possible range 0–18)	26	5.3 (4.0)	21	4.1 (3.0)	18	0.94 (2.62)	0.36	-0.12 to 0.83
Health status: EQ-5D-5L index value (possible range 0–1)	29	0.6 (0.2)	21	0.5 (0.3)	21	0.13 (0.20)	0.67	0.19 to 1.14
Health status: EQ-VAS (possible range 0–100)	29	66.3 (25.8)	21	65.0 (21.7)	21	4.86 (19.55)	0.25	-0.19 to 0.68
Disease-related functioning: ALS FRS-R (possible range 0–48)	29	35.2 (7.6)	21	30.9 (8.1)	21	4.52 (6.65)	0.68	0.20 to 1.15
Psychological flexibility: AAQ-II (possible range 7–49)	29	17.2 (8.5)	21	17.2 (7.8)	21	-0.52 (8.51)	-0.06	-0.49 to 0.37
Treatment satisfaction: STTS-R								
Satisfaction with therapy (possible range 6–30)	-	-	19	24.5 (5.0)	-	-	-	-
Satisfaction with therapist (possible range 6–30)	-	-	19	28.1 (2.3)	-	-	-	-
Global improvement (possible range 1–5)	-	-	19	2.0 (0.7)	-	-	-	-
*Caregivers (N* = *18)*								
Health status: EQ-5D-5L index value (possible range 0–1)	17	0.8 (0.3)	9	0.9 (0.1)	8	-0.02 (0.07)	-0.34	-1.04 to 0.39
Health status: EQ-VAS (possible range 0–100)	17	77.4 (17.4)	9	84.0 (14.8)	8	1.75 (10.63)	0.17	-0.54 to 0.86
Caregiver burden: ZBI (possible range 0–88)	17	18.9 (14.0)	9	19.2 (15.1)	8	-8.50 (11.30)	-0.75	-1.53 to 0.06

One participant who was recruited but later found to be ineligible is not included here

*AAQ-II* Acceptance and Action Questionnaire-II: higher scores indicate greater psychological inflexibility, *ALS FRS-R* Amyotrophic Lateral Sclerosis Functional Rating Scale-Revised: higher scores indicate better disease-related functioning, *CI* Confidence interval, *EQ-5D-5L* Higher scores indicate better health status, *EQ-VAS* EQ-Visual Analogue Scale: higher scores indicate better health status, *ES* Effect size, *mHADS* Hospital Anxiety and Depression Scale modified for plwMND such that one depression item and one anxiety item were not scored, as previously recommended [43]: higher scores indicate greater depression or anxiety, *MQOL-R* McGill Quality of Life Questionnaire-Revised: higher scores indicate better quality of life, *SD* standard deviation, *STTS-R* Satisfaction with Therapy and Therapist Scale-Revised: higher scores indicate greater satisfaction with therapy or the therapist; higher scores for global improvement indicate higher perceived improvement, *ZBI* Zarit Burden Interview: higher scores indicate higher caregiver burden

^a^One depression item and one anxiety item were not scored on the HADS, as previously recommended [43]

#### Feasibility

Eighteen percent (29/159) of potential participants who were screened and eligible were recruited. The majority of potential participants who were screened were not recruited for feasibility reasons, including ineligibility (48%, 62/129) and declining consent or uncontactable (14%, 18/129) (see Fig. 1). Only 14% (4/29) of recruited participants were lost to follow-up for feasibility reasons (death, physical health deterioration or hospital appointments).

#### Patient- and caregiver-reported outcomes

As no statistical analyses were conducted following previous recommendations [49, 50], changes in outcomes are presented descriptively. Data were suggestive of small improvements in anxiety and depression (mHADS) and non-physical quality of life (MQOL-R) from baseline to 6 months in plwMND (see Table 4). This was despite a small but expected deterioration in disease-related functioning (ALS FRS-R), health status (EQ-5D-5L) and physical quality of life (MQOL-R). There was no change in psychological flexibility (AAQ-II).

Table 5 presents the number of plwMND who demonstrated reliable improvement or deterioration on outcome measures at 6 months. Most notably, reliable improvement in anxiety (mHADS) and psychological quality of life (MQOL-R) was observed in three participants (17%), and was also seen for depression (mHADS) and psychological flexibility (AAQ-II) in one participant (6%). Only a small number of participants showed reliable deterioration in psychological quality of life (MQOL-R, *N* = 2, 10%) and psychological flexibility (AAQ-II, *N* = 3, 14%), while none showed reliable deterioration in anxiety or depression (mHADS). In contrast, but as expected with a neurodegenerative disease, nine participants (43%) showed reliable deterioration in disease-related functioning (ALS FRS-R) and six (29%) demonstrated reliable deterioration in health status (EQ-5D-5L). However, this was not mirrored in physical quality of life (MQOL-R), most likely due to the poorer internal consistency of this sub-scale (Cronbach's alpha = 0.66) [41]. Perhaps not surprisingly given the varied pattern of results, the number of participants demonstrating reliable improvement or deterioration in overall quality of life (MQOL-R) was mixed, with three participants (14%) demonstrating reliable improvement and four (19%) showing reliable deterioration.

**Table 5:** Reliable change in plwMND and caregivers from baseline to 6 months

Outcome measure	N	Cronbach's alpha (study ref. no.)	Reliable deterioration (N)	No reliable change (N)	Reliable improvement (N)
*plwMND*					
Quality of life: MQOL-R					
Global^a^	21	N/A	N/A	N/A	N/A
Physical	21	0.66 [41]	0	21	0
Psychological	21	0.85 [41]	2	16	3
Existential	21	0.78 [41]	0	21	0
Social	21	0.87 [41]	1	19	1
Total score	21	0.94 [41]	4	14	3
Mood: mHADS^b^					
Depression	18	0.82 [53]	0	17	1
Anxiety	18	0.83 [53]	0	15	3
Health status: EQ-5D-5L index value	21	0.83 [55]	6	15	0
Health status: EQ-VAS^a^	21	N/A	N/A	N/A	N/A
Disease-related functioning: ALS FRS-R	21	0.88 [54]	9	11	1
Psychological flexibility: AAQ-II	21	0.84 [44]	3	17	1
*Caregivers*					
Health status: EQ-5D-5L index value	8	0.82 [56]	0	7	1
Health status: EQ-VAS^a^	8	N/A	N/A	N/A	N/A
Caregiver burden: ZBI	8	0.93 [57]	3	5	0

One participant who was recruited but later found to be ineligible is not included here

*AAQ-II* Acceptance and Action Questionnaire-II, *ALS FRS-R* Amyotrophic Lateral Sclerosis Functional Rating Scale-Revised, *CI* Confidence interval, *EQ-5D-5L. EQ-VAS* EQ-Visual Analogue Scale, *ES* Effect size, *mHADS* Hospital Anxiety and Depression Scale modified for plwMND such that one depression item and one anxiety item were not scored, as previously recommended [43], *MQOL-R* McGill Quality of Life Questionnaire-Revised, *SD* Standard deviation, *STTS-R* Satisfaction with Therapy and Therapist Scale-Revised, *ZBI* Zarit Burden Interview

^a^Measures of internal consistency do not apply to single-item measures

^b^One depression item and one anxiety item were not scored on the HADS, as previously recommended [43]

As shown in Table 6, the proportion of plwMND meeting case levels on the mHADS was smaller at 6 months compared to baseline for both anxiety (baseline: 4/26, 15%; 6 months: 2/21, 10%) and depression (baseline: 3/26, 12%; 6 months: 1/21, 5%).

**Table 6:** Case levels of anxiety and depression in plwMND at baseline and 6 months

Hospital Anxiety and Depression Scale modified for plwMND^a^	Baseline N	6 months N
Anxiety (possible range 0–18)^b^		
Case (score ≥ 9)	4	2
Borderline (score 7–8)	6	2
Non-case (score ≤ 6)	16	17
Missing	3	8
Depression (possible range 0–18)^b^		
Case (score ≥ 8)	3	1
Borderline (score 5–7)	3	2
Non-case (score ≤ 4)	20	18
Missing	3	8

One participant who was recruited but later found to be ineligible is not included here

^a^One anxiety item and one depression item were not scored on the HADS due to confounding with MND symptoms, as previously recommended [43]

^b^Recommended MND-specific scoring cut-offs for anxiety and depression are based on a Rasch analysis [43]

With respect to caregivers, data suggested a small improvement in health status on the EQ-5D-5L from baseline to 6 months (see Table 4), with one participant (13%) demonstrating reliable improvement at 6 months (see Table 5). This was observed alongside a small increase in caregiver burden on the ZBI, with three participants (38%) demonstrating reliable deterioration at 6 months.

#### Adverse events

There were two reports of non-physical adverse events and two of serious adverse events. None were deemed to be related to the intervention by the Study Steering Committee.

### Treatment fidelity

High rates of overall adherence to the manual (mean 4.9, SD 0.2) and overall ACT competence of therapists (mean 4.7, SD 0.5) were observed using the ACT-TICM. Furthermore, there was no evidence of ACT-inconsistent items in any of the rated sessions (mean 1.0, SD 0.0).

## Discussion

This study showed that it is feasible to recruit plwMND to an uncontrolled study of ACT for improving psychological health and this type of intervention is acceptable to this population. A priori indicators of success with respect to uptake and initial engagement with therapy were met. Feasibility and acceptability of the intervention were further supported by secondary outcomes, including satisfaction with therapy and attrition rate. These indicated good evidence of acceptability and feasibility. Data were also suggestive descriptively of small improvements in outcome measures from baseline to 6 months in plwMND – most notably, anxiety and psychological quality of life, which were reliably observed in 17% of participants. This was despite a small but expected deterioration in disease-related functioning and health status from baseline to 6 months, which were reliably observed in 29–43% of participants. It is important to note that, as required by the Funder (NIHR)'s commissioned call [39], plwMND were not recruited to this feasibility study on the basis of psychological distress. Furthermore, ACT is aimed at increasing life-enriching activities, alongside difficult thoughts and emotions, rather than symptomatic reduction. Therefore, small rather than large changes in psychological distress across time might be expected in this population.

As there was no control group in the current study, these small changes could simply be a product of the disease process, a higher rate of missing outcome data at 6 months or chance observations given the small sample size. Furthermore, it is not possible to determine whether results reflect an ineffective treatment, beneficial effects being countered by deterioration due to disease progression or a possible stabilisation of these outcomes across time. In support of the latter interpretations, these results are consistent with a previous RCT of meditation training compared to usual care in plwMND [21]. This RCT reported that quality of life, depression and anxiety remained stable from baseline to 12 months in the meditation arm, but declined across time in the usual care arm. A similar pattern of stabilisation of quality of life, anxiety and depression in the treatment group compared to deterioration in the control group was recently reported in a small non-randomised controlled trial of empathy-based supportive counselling for people with ALS [62]. In contrast, a small RCT of a non-meditative mindfulness intervention vs. a wait-list control for people with ALS reported stabilisation of quality of life, depression and anxiety in the control arm, but improvement in these measures in the mindfulness arm [63]. However, these findings were limited by a high attrition rate and small sample size as 47% of participants (22/47) dropped out by 6-month follow-up. These potentially conflicting results indicate that future research should seek to evaluate the clinical effectiveness of ACT adapted for plwMND in comparison to a control arm in a fully powered RCT.

We found minimal changes across time on the AAQ-II, the most common ACT process measure of psychological flexibility. Although this might indicate that the intervention resulted in little change in ACT core processes, both the construct and discriminant validity of the AAQ-II have been questioned [64–66]. In particular, it has been suggested that although the AAQ-II mainly measures psychological inflexibility, it is contaminated with distress content [64–66], and has been shown to be prone to comprehension errors in clinical populations [67]. Consequently, future studies of ACT interventions for plwMND should consider using alternative measures of psychological flexibility such as the Comprehensive Assessment of ACT processes [68] or the Multidimensional Psychological Flexibility Inventory [69].

Twenty-eight percent (8/29) of participants were lost to follow-up in the current study, which was higher than anticipated (20% at 6-months) [58]. Reassuringly, few participants dropped out due to a lack of acceptability (2/29, 7%), with the remainder being due to feasibility issues (such as death and heath deterioration). Although the attrition rate was higher than anticipated, it is important to view this in the context of rates observed in other studies of psychological interventions for plwMND. For example, an attrition rate of 57% by 6 months was reported in an RCT of meditation training, with disease progression and death being given as reasons for drop out [21]. This suggests that ways to reduce drop out due to feasibility issues need to be carefully considered in any future RCTs of psychological interventions for plwMND. Possible solutions include: i) limiting the duration of follow-up (e.g. to 9 rather than 12 months post-baseline); ii) inflating the sample size to ensure maintenance of power despite drop out; and iii) excluding those in stages 4A/4B of the King’s College London clinical staging system [32], as these are markers of significantly reduced life expectancy and more advanced disease stage (and hence an indicator that participants might not survive the duration of the RCT). The fact that 39% (62/159) of potential participants were not eligible at screening in this study, mainly due to the use of non-invasive ventilation/percutaneous endoscopic gastrostomy, suggests that using the clinical staging system to reduce drop out would need to be carefully balanced with ensuring recruitment remained feasible in any future RCT.

To our knowledge, this is the first study of the acceptability, feasibility, and preliminary estimates of the effectiveness of ACT adapted for plwMND. However, there are several limitations. First, the majority of participants self-identified as White/White British and so results cannot be generalised to a broader population with MND. Second, the number of participants scoring in the clinical range for depression and anxiety at baseline (12% and 15%, respectively) and the median number of years following symptom onset (2.3 years) suggest that the sample might not be representative of all plwMND seen in MND clinics. Third, by virtue of its design, this feasibility study was not adequately powered to examine clinical effectiveness, and findings are therefore reported descriptively rather than statistically, as recommended [49, 50]. Fourth, as participants were only followed up for 6 months, it is uncertain whether any possible stabilisation of psychological quality of life or mood was maintained beyond 6 months or whether any gains were made beyond this timepoint. Fifth, plwMND who had a need for gastrostomy feeding or non-invasive ventilation were excluded from the study in order to reduce potential attrition. Therefore, it is unclear whether ACT is beneficial for those in a more advanced disease stage. Future studies should consider ways of examining the potential effectiveness of ACT (and other psychological therapies) across the MND disease course, while at the same time minimising attrition. Finally, as noted, the lack of a control group limits the interpretation of findings. For example, the potentially smaller proportion of plwMND meeting case levels of anxiety and depression at 6-months may be due to non-specific therapeutic factors such as social support or spontaneous recovery. Therefore, descriptive results pertaining to the preliminary effectiveness of ACT for plwMND should be interpreted with caution.

## Conclusions

There was good evidence of the acceptability and feasibility of ACT for plwMND, in addition to possible signals of efficacy, particularly with respect to anxiety and psychological quality of life. However, limitations included the lack of control group and small sample size. Consequently, a fully powered RCT evaluating the clinical and cost-effectiveness of ACT adapted specifically for plwMND is currently underway [70].

## Supplementary Information

**Additional file 1.** The consolidated standards of reporting trials (CONSORT) checklist (extension for randomised pilot or feasibility trials).

**Additional file 2.** The template for intervention description and replication (TIDieR) checklist.

**Additional file 3.** Information about baseline measures and outcome measures.

**Additional file 4.** Psychologically inflexible processes and their psychologically flexible counterparts, with examples relevant to plwMND.

## Data Availability

The dataset used and/or analysed during the current study is available from the corresponding author upon reasonable request.

## Abbreviations

AAQ-II: Acceptance and Action Questionnaire-II
ACT: Acceptance and Commitment Therapy
ACT-TICM: ACT Treatment Integrity Coding Manual
ALS: Amyotrophic Lateral Sclerosis
ALS FRS-R: ALS Functional Rating Scale-Revised
CSRI: Client Service Receipt Inventory
CONSORT: Consolidated Standards of Reporting Trials
ECAS: Edinburgh Cognitive Behavioural ALS Screen
IQR: Interquartile range
mHADS: Hospital Anxiety and Depression Scale modified for plwMND
MiND-B: Motor Neuron Disease Behavioural Instrument
MND: Motor Neuron Disease
MQOL-R: McGill Quality of Life Questionnaire-Revised
NIHR: National Institute for Health and Care Research
plwMND: People living with MND
RCI: Reliable Change Index
RCT: Randomised controlled trial
SD: Standard deviation
STTS-R: Satisfaction with Therapy and Therapist Scale-Revised
TIDieR: Template for Intervention Description and Replication
VAS: Visual Analogue Scale
ZBI: Zarit Burden Interview
