# Supplementary material for: Acceptance and Commitment Therapy for people living with motor neuron disease: an uncontrolled feasibility study
Source: Pilot Feasibility Stud. 2023 Jul 7;9:116. doi: 10.1186/s40814-023-01354-7 (PMC10327371; doi:10.1186/s40814-023-01354-7)
Supplement: Supplementary file 3 — Additional file 3. Information about baseline measures and outcome measures. [file 40814_2023_1354_MOESM3_ESM.docx]

Additional File 3: Information about baseline measures and outcome measures.

## Baseline measures and outcome measures

Further details about baseline measures and primary and secondary outcome measures are provided below:

### plwMND

The Edinburgh Cognitive Behavioural ALS Screen (ECAS) (1) and the Motor Neuron Disease Behavioural Instrument (MiND-B) (2) were used at baseline to assess cognitive and behavioural functioning in plwMND. The ECAS is a brief screening measure of cognitive and behavioural functioning in plwMND. It assesses cognition in five domains (language, verbal fluency, executive functioning, memory and visuospatial functioning), in addition to frontal behavioural symptoms. The MiND-B is a brief screening measure of behavioural difficulties in plwMND that assesses behavioural symptoms in three domains (apathy, disinhibition and stereotypical behaviour).

The following outcome measures were assessed at baseline and 6 months, unless otherwise stated:

a) McGill Quality of Life Questionnaire-Revised (3): A 17-item self-report global measure of quality of life that has been validated in plwMND (4,5). A single item measures overall quality of life, while 4 subscales measure quality of life in Physical, Psychological, Existential and Social domains. A total score is calculated from the mean of the 4 subscale scores. Higher scores indicate better quality of life;

b) Hospital Anxiety and Depression Scale (6): A 14-item self-report measure of anxiety and depression that provides separate scores for depression and anxiety. Following validation in plwMND (7), it is recommended that one item on the depression scale that assesses psychomotor retardation and one item on the anxiety scale that assesses restlessness are omitted from analyses as these overlap with physical symptoms of MND. Higher scores indicate greater depression or anxiety;

c) Acceptance and Action Questionnaire-II (8): A 7-item self-report process measure of psychological flexibility or experiential avoidance that is commonly used in ACT trials. Higher scores indicate greater psychological inflexibility;

d) EQ-5D-5L (9): A 5-item self-report measure of health status, used to calculate utility scores for use in economic evaluations, including in plwMND. Each of the 5 items is rated on a 5-point scale from no problems to extreme problems, and scores are converted into a single index value ranging from 0 to 1, where higher scores indicate better health status. A single-item assesses health status on a visual analogue scale from 0 (worst health you can imagine) to 100 (best health you can imagine). Higher scores indicate better health status;

e) Amyotrophic Lateral Sclerosis Functional Rating Scale-Revised (10): A 12-item measure of function developed for plwMND that can be used as an indicator of disease progression. Higher scores indicate better disease-related functioning;

f) Client Service Receipt Inventory (11) modified for plwMND: A measure of service utilisation used to calculate costs, that was modified for use with plwMND so that it measured costs associated with health and social care, equipment and home adaptations.

g) Satisfaction with Therapy and Therapist Scale-Revised (12) at 6 months only: A 12-item self-report questionnaire with two sub-scales measuring satisfaction with therapy and satisfaction with the therapist. Each item is rated on a scale from 1 (strongly disagree) to 5 (strongly agree), with the exception of a single item assessing perceived global improvement that is rated on a scale from 1 ("made things a lot better") to 5 ("made things a lot worse"). Higher scores indicate greater satisfaction with therapy or the therapist. There is no set definition of what constitutes “satisfactory” on this questionnaire and so this was defined as a total score of 21 or more on the Satisfaction with Therapy subscale.

### Caregivers

a) EQ-5D-5L: See above;

b) Zarit Burden Interview (13): A well-validated 22-item self-report measure of caregiver burden. Higher scores indicate higher caregiver burden. This measure was included since supporting plwMND to engage in ACT could place extra burden on caregivers.

# References

1. Niven E, Newton J, Foley J, Colville S, Swingler R, Chandran S, et al. Validation of the Edinburgh Cognitive and Behavioural Amyotrophic Lateral Sclerosis Screen (ECAS): A cognitive tool for motor disorders. Amyotroph Lateral Scler Front Degener. 2015 Apr 27;16(3–4):172–9.
2. Mioshi E, Hsieh S, Caga J, Ramsey E, Chen K, Lillo P, et al. A novel tool to detect behavioural symptoms in ALS. Amyotroph Lateral Scler Front Degener. 2014 Jun;15(3–4):298–304.
3. Cohen SR, Sawatzky R, Russell LB, Shahidi J, Heyland DK, Gadermann AM. Measuring the quality of life of people at the end of life: The McGill Quality of Life Questionnaire-Revised. Palliat Med. 2017;31(2):120–9.
4. Simmons Z, Bremer BA, Robbins RA, Walsh SM, Fischer S. Quality of life in ALS depends on factors other than strength and physical function. Neurology. 2000 Aug 8;55(3):388–92.
5. Robbins RA, Simmons Z, Bremer BA, Walsh SM, Fischer S. Quality of life in ALS is maintained as physical function declines. Neurology. 2001 Feb 27;56(4):442–4.
6. Zigmond AS, Snaith RP. The Hospital Anxiety and Depression Scale. Acta Psychiatr Scand. 1983 Jun;67(6):361–70.
7. Gibbons CJ, Mills RJ, Thornton EW, Ealing J, Mitchell JD, Shaw PJ, et al. Rasch analysis of the hospital anxiety and depression scale (hads) for use in motor neurone disease. Health Qual Life Outcomes. 2011;9(1):82.
8. Bond FW, Hayes SC, Baer RA, Carpenter KM, Guenole N, Orcutt HK, et al. Preliminary Psychometric Properties of the Acceptance and Action Questionnaire–II: A Revised Measure of Psychological Inflexibility and Experiential Avoidance. Behav Ther. 2011 Dec 1;42(4):676–88.
9. Herdman M, Gudex C, Lloyd A, Janssen M, Kind P, Parkin D, et al. Development and preliminary testing of the new five-level version of EQ-5D (EQ-5D-5L). Qual Life Res Int J Qual Life Asp Treat Care Rehabil. 2011 Dec;20(10):1727–36.
10. Cedarbaum JM, Stambler N, Malta E, Fuller C, Hilt D, Thurmond B, et al. The ALSFRS-R: a revised ALS functional rating scale that incorporates assessments of respiratory function. J Neurol Sci. 1999 Oct;169(1–2):13–21.
11. Beecham J, Knapp M. Costing psychiatric interventions. In: Thornicroft G, Brewin C, Wing J, editors. Measuring Mental Health Needs [Internet]. London, UK: Gaskell; 2001 [cited 2020 Jul 14]. p. 200–24. Available from: <http://www.rcpsych.ac.uk>.
12. Oei TPS, Green AL. The Satisfaction With Therapy and Therapist Scale--Revised (STTS-R) for group psychotherapy: Psychometric properties and confirmatory factor analysis. Prof Psychol Res Pract. 2008;39(4):435–42.
13. Zarit SH, Reever KE, Bach-Peterson J. Relatives of the Impaired Elderly: Correlates of Feelings of Burden. The Gerontologist. 1980 Dec 1;20(6):649–55.
