# Supplementary material for: Acceptance and Commitment Therapy for people living with motor neuron disease: an uncontrolled feasibility study
Source: Pilot Feasibility Stud. 2023 Jul 7;9:116. doi: 10.1186/s40814-023-01354-7 (PMC10327371; doi:10.1186/s40814-023-01354-7)
Supplement: Supplementary file 4 — Additional file 4. Psychologically inflexible processes and their psychologically flexible counterparts, with examples relevant to plwMND. [file 40814_2023_1354_MOESM4_ESM.docx]

Additional File 4. Psychologically inflexible processes and their psychologically flexible counterparts, with examples relevant to plwMND.

| **Psychologically inflexible process** | **Psychologically flexible process** | **Examples of psychologically inflexible processes in a person living with MND** |
| --- | --- | --- |
| *Experiential avoidance:* Trying to control, avoid, get rid of or change the frequency or form of unwanted internal experiences (e.g. thoughts, images, memories, emotions, sensations). | *Acceptance:* Reducing avoidance of or opening up to unwanted internal experiences (when this might be a barrier to life enriching activity), particularly when this serves one’s goals and values. | Avoiding going to MND support groups, avoiding using a wheelchair, avoiding planning for future treatment or avoiding thinking about end-of-life issues. |
| *Cognitive fusion:* Getting hooked by or fused with negative or unhelpful thoughts, images or memories, or acting as if these internal experiences are literally true. | *Defusion:* Unhooking or stepping back from negative or unhelpful thoughts, images or memories, or seeing these internal experiences for what they are (e.g. just thoughts). | Fusion with thoughts such as “What’s the point…?”, “It’s not the same” or “It’s not fair”. |
| *Dominance of past and future:* Being stuck in one's head, ruminating about the past or worrying about the future. | *Contact with the present moment:* Being aware of moment to moment experiences in the “here and now”. | Worrying about the future (e.g. about the prognosis or disease progression) or ruminating about the past (e.g. who they used to be and how things used to be). |
| *Self-as-content: S*eeing oneself as the content of one's internal experiences, or being caught up in or attached to the labels or stories that one tells about oneself. | *Self-as-context:* Seeing oneself as distinct from the content of one's internal experiences rather than being defined by them, or holding one’s labels and stories lightly. | Strong attachment to labels or self-stories such as “I’m not the person I used to be” or “I was always the strong one”. |
| *Lack of clarity or loss of contact with values:* Losing connection with or not knowing what really matters to oneself (i.e. what is important and meaningful). | *Values:* Knowing what really matters to oneself in one's life and acting accordingly. | Unable to identify what is important or what matters to them (e.g. “I don’t know anymore”). |
| *Inaction, impulsivity or avoidant persistence:* Failing to act in accordance with what really matters to oneself through avoidance or inaction. | *Committed action:* Planning or choosing what one will do, doing it, and persisting in it or changing it, in line with personally meaningful goals and values. | Lack of engagement in valued activities or living a life in service of avoidance rather than values. |
